# Indigestion

**Published:** 1889-01

**Authors:** 


### INDIGESTION.

Indigestion is something more than simply an inconvenience. A body which is served with food by a dyspeptic stomach, receives very poor material of which to rebuild its tissues. None of the food is perfectly digested, and hence the quality of all the tissues is deteriorated. Besides this, the septic changes which take place in the stomach and bowels, produce various poisonous substances, which are absorbed along with the food, and which poison and irritate the brain and nerves, and produce various disorders and discomforts which are ofttimes attributed to other causes. Even the imperfectly digested food is treated by the system as waste or poisonous material, and instead of being used to repair the wastes of the body, is excreted, or thrown off by the liver and kidneys, with the waste elements of the system.

The stomach sometimes holds up wonderfully under the heavy burdens laid upon it, and digests a much larger amount of food than is necessary to supply the wants of the body. In such cases, the excessive amount of nutriment received is either at once excreted, or accumulates in the tissues, clogging the various organs, and interfering with their proper activity. Accumulations of this sort are the chief cause of gout, rheumatism, biliousness, and numerous other disorders which are usually attributed to other causes.

Eating when tired, and engaging in active mental or physical exercise immediately after a hearty meal, are two of the most common sins against dietetic rectitude in our modern civilization. An old medical writer tells us that a hundred years ago it was the custom among the merchants of Edinburg to take two hours' "nooning" for dinner in the middle of the day, during which time the shops were closed, and all business suspended. It is quite hopeless to attempt a resurrection of this good old-fashioned custom in these fast times; and the best thing we can suggest is that no hearty meal should be eaten during the active business hours of the day, unless at least an hour or two can be allowed after the meal has been taken, to give the stomach opportunity to get the digestive process well under way. The plan which our personal experience leads us to prefer is to defer the hearty meal, as did the old Romans, until the latter part of the day, say four o'clock in the afternoon, taking if necessary, an apple, a bunch of grapes, an orange or two, or some equally simple food at midday, to appease the the clamoring of the stomach, until it has become accustomed to the lengthened interval between the first and second meals. Two meals a day are in every way preferable to a larger number. The ancient Greeks and Romans took but one meal *per diem.*. During the republican era, the Roman custom was to eat twice a day, breakfast being simply a light repast of fruit and bread. At the present time, the two-meals-a-day plan prevails quite extensively in France and Spain, especially among the better classes. The inmates of the hospitals in Paris are supplied with but two meals a day. The same is true respecting the soidiers of the French army.—*Good Health.*

